# Sirtuin 6 promotes eosinophil differentiation by activating GATA‐1 transcription factor

**DOI:** 10.1111/acel.13418

**Published:** 2021-06-14

**Authors:** In Hyuk Bang, Dami Park, Youngyi Lee, Hwangeui Cho, Byung‐Hyun Park, Eun Ju Bae

**Affiliations:** ^1^ Department of Biochemistry and Molecular Biology Chonbuk National University Medical School Jeonju Korea; ^2^ College of Pharmacy Chonbuk National University Jeonju Korea

**Keywords:** acetylation, adipocyte beiging, eosinophil, GATA‐1, M2 macrophage, p300, Sirt6

## Abstract

There is evidence emerging that exposure to cold temperatures enhances alternative activation of macrophages in white adipose tissue (WAT), which promotes adipocyte beiging and adaptive thermogenesis. Although we recently reported that NAD^+^‐dependent deacetylase sirtuin 6 (Sirt6) drives alternatively activated (M2) macrophage polarization, the role of myeloid Sirt6 in adaptive thermogenesis had remained elusive. In this study, we demonstrate that myeloid Sirt6 deficiency impaired both thermogenic responses and M2 macrophage infiltration in subcutaneous WAT (scWAT) during cold exposure. Moreover, the infiltration of Siglec‐F‐positive eosinophils in scWAT and Th2 cytokines levels was reduced in myeloid Sirt6 knockout mice. An *ex vivo* bone marrow‐derived cell culture experiment indicated that Sirt6 was required for eosinophil differentiation independent of its deacetylase activity. Data from our in vitro experiments show that Sirt6 acted as a transcriptional cofactor of GATA‐1, independent of its catalytic function as a deacetylase or ADP‐ribosyltransferase. Specifically, Sirt6 physically interacted with GATA‐1, and enhanced GATA‐1’s acetylation and transcriptional activity by facilitating its cooperation with p300. Overall, our results suggest that myeloid Sirt6 plays an important role in eosinophil differentiation and fat beiging/adaptive thermogenesis, which is at least in part due to its ability to bind GATA‐1 and stimulate its transcriptional activity.

## Graphical abstract

Eosinophil infiltration into white adipose tissue has been found to be crucial for M2 macrophage polarization and adipose tissue browning upon cold exposure. We identify Sirt6 as an important regulator of eosinophil differentiation and thus adaptive thermogenesis by enhancing the acetylation and transcriptional activity of GATA‐1 by facilitating its cooperation with p300 acetyltransferase.

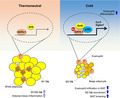

## 1 INTRODUCTION

Rodents and humans housed at above thermoneutral temperature do not normally require non‐shivering or adaptive thermogenesis to maintain their body temperature. However, when chronically housed at below thermoneutral temperature or after acute cold exposure they generate heat through mitochondrial uncoupling protein 1 (UCP1)‐mediated thermogenesis in brown adipose tissue (BAT) (Chouchani et al., 2019). Additionally, cold stimuli induce browning of white adipose tissue (WAT), producing beige adipocytes, mainly in subcutaneous WAT *via* adrenergic stimulation (Huang et al., 2017; Jimenez et al., 2003). BAT is formed during embryonic development but browning of WAT is highly inducible, occurring only in response to cold stimuli or other specific circumstances such as calorie restriction, exercise, metabolic surgery, or intestinal microbiota depletion (Fabbiano et al., 2016; Neinast et al., 2015; Suarez‐Zamorano et al., 2015). Both human and animal studies suggest that BAT and adaptive thermogenic activity correlate inversely with WAT mass and related insulin resistance (van Marken Lichtenbelt et al., 2009; Virtanen et al., 2009). It is noteworthy that abundant beige adipocytes have been observed in BAT in adult humans and that human UCP1‐positive adipocytes have similar molecular features with beige fat in mice (Wu et al., 2012). Thus, beige adipocytes have attracted considerable attention as a cellular target against obesity, particularly in those who have only a small amount of BAT such as elderly or obese people.

Crosstalk between white adipocytes and infiltrating immune cells is well established as a pathogenesis of obesity‐linked tissue inflammation and consequent insulin resistance. Likewise, the tight interplay among various immune cells is necessary for a proper browning of adipocytes and metabolic homeostasis. These cells include macrophages, eosinophils, group 2 innate lymphoid cells (ILC2), regulatory T cells, and T helper 2 (Th2) cells (Hui et al., 2015; Kalin et al., 2017; Qiu et al., 2014; Villarroya et al., 2018). Accumulating evidence indicates that alternatively activated M2 macrophages are the main actors driving WAT browning (Hui et al., 2015; Nguyen et al., 2011; Qiu et al., 2014). Mechanistically, upon cold exposure, eosinophils and ILC2 recruited into WAT produce Th2 cytokines such as interleukin (IL)‐4, ‐5, and ‐13, which in turn stimulate M2 polarization of macrophages. M2 macrophages were initially reported to express tyrosine hydroxylase, a rate‐limiting enzyme for catecholamine synthesis, and to produce norepinephrine, thus facilitating browning of white adipocytes (Nguyen et al., 2011). This finding has since been challenged by other groups, however, who have shown that M2 macrophages do not synthesize norepinephrine (Fischer et al., 2017). Instead, catecholamine catabolism has been implicated as affecting sympathetic tone in WAT (Pirzgalska et al., 2017; Song et al., 2019). Although the detailed mechanism of M2 macrophage‐mediated WAT browning remains unclear, the importance of type 2 cytokine signaling and M2 macrophages in WAT browning is nonetheless significant, as revealed by the study in which ablation of the IL‐4 receptor in myeloid impaired thermogenic activity in mice (Nguyen et al., 2011).

Sirtuins (Sirt1‐7) are highly conserved NAD^+^‐dependent lysine deacetylases/deacylases crucial for cellular homeostasis. Sirt6 is a nuclear sirtuin with ADP‐ribosyltransferase activity in addition to deacetylase activity (Liszt et al., 2005; Mao et al., 2011; Michishita et al., 2008). As a regulator of gene expression, Sirt6 has been implicated in inflammation and metabolism and emerged as a therapeutic target in metabolic diseases (Lee et al., 2017; Michishita et al., 2008; Moon et al., 2019; Woo et al., 2018). Three findings of our observation indicate that Sirt6 is a central actor in determining macrophage phenotype: (1) on the one hand, increased M1 polarization in myeloid Sirt6 knockout (mS6KO) mice fed a high‐fat diet (HFD) increases adipose tissue inflammation and insulin resistance (Lee et al., 2017); (2) on the other hand, suppression of M2 polarization in the same mice is associated with delayed tissue repair (Koo et al., 2019); (3) moreover, genetic deletion of Sirt6 in adipocytes was found to trigger M2‐to‐M1 transition of macrophages by suppressing IL‐4 production in adipocytes (Song et al., 2019). Taking these findings into consideration, we were interested in exploring whether myeloid Sirt6 impacts browning of WAT and adaptive thermogenesis. Accordingly, this study investigated the metabolic phenotypes of mS6KO mice in response to cold stress, and their underlying mechanism. Our results show that myeloid Sirt6 is a critical determinant of eosinophil differentiation and M2 macrophage polarization for WAT browning and cold tolerance.

## 2 RESULTS

### 2.1 Sirt6 expression is selectively induced in scWAT and BAT by cold exposure

To explore the adipose depot‐specific differential role of Sirt6 in cold‐induced adaptive thermogenesis, we measured the Sirt6 expression in different adipose tissues in C57BL/6J male mice. Consistent with previous reports (Yao et al., 2017), western blotting analysis revealed substantial induction of Sirt6 by cold in scWAT as well as in BAT, although no change was observed in eWAT (Figure S1a). Interestingly, the cold‐induced increase in Sirt6 in scWAT occurred specifically in SVCs rather than adipocytes (Figure S1b). On the contrary, cold‐induced UCP1 expression was much higher in the mature adipocytes fraction (Figure S1b). From these results, it can be inferred that Sirt6 in myeloid cells may play an important role in fat beiging or UCP1 induction in adipocytes.

### 2.2 Myeloid‐specific Sirt6 deficiency leads to impairment thermogenesis and WAT beiging in mice

**FIGURE 1:**
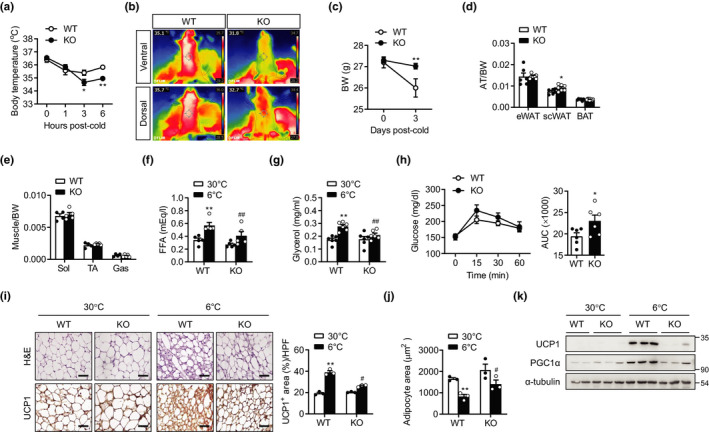
Impairment of adaptive thermogenesis and subcutaneous fat beiging in mS6KO mice after cold exposure. Eight‐week‐old male mS6KO mice and their wild‐type (WT) littermates were housed at 30°C for 1 day and then exposed to cold (6°C). (a) Rectal temperature of mice was measured at indicated time points after cold exposure (*n* = 7–8). (b) Representative infrared images showing mS6KO and WT mice housed at 6°C for 3 days. (c) Body weights in WT and mS6KO mice housed at 6°C for 3 days (*n* = 7–8). (d, e) Mice were killed after 3 days of cold exposure. Weights of adipose tissue(AT), soleus (Sol), tibialis anterior (TA), and gastrocnemius (Gas) muscles normalized to BW are presented (*n* = 6). (f, g) Serum levels of free fatty acid (FFA) and glycerol were measured (*n* = 4–6). (h) A glucose tolerance test was performed after 3‐day cold exposure and areas under the curve (AUC) of blood glucose were compared (*n* = 6). (i) scWAT was H&E stained or immunostained with an antibody against UCP1. Bars = 25 μm. The percentage of UCP1‐positive area was quantified in three high‐power fields (HPF) per mouse. The graph shows quantification results (*n* = 3). (j) Cell size analysis of adipocytes from scWAT was carried out on two sections per mouse. (k) Protein levels of thermogenic markers were determined by western blotting. Data represent the mean ±SEM. *p < 0.05 and **p < 0.01 vs. WT 30°C; ^#^ *p* < 0.05 and ^##^ *p* < 0.01 vs. WT 6°C

Because we observed a selective and marked induction of Sirt6 expression in SVCs of scWAT (Figure S1b), we generated mS6KO mice and exposed them to cold (6°C) for 3 days. We found that male mS6KO mice showed a significantly lower rectal temperature than wild type (WT) mice after 3‐ and 6 h‐cold exposure (Figure 1a). We further monitored the body surface temperatures in the ventral and dorsal areas after cold exposure using infrared imaging. Consistent with the lower rectal temperatures, decreased heat dissipation was observed in the mS6KO mice compared with the WT mice (Figure 1b). Accordingly, body weight after 3‐day cold exposure was higher in the mS6KO mice compared with the WT mice (Figure 1c) without changes either in basal body weights (30°C) or in food intake (data not shown). We then compared the adipose tissue and skeletal muscle weights normalized to body weights and found that scWAT weight only was significantly higher in the male mS6KO mice compared with the WT mice, whereas those of BAT, eWAT, and skeletal muscle did not differ between genotypes (Figure 1d,e). Because cold exposure stimulates triglyceride (TG) lipolysis to release free fatty acids (FFA) and glycerol from adipocytes *via* adrenergic signaling (Nguyen et al., 2011), we measured circulating FFA and glycerol. Results revealed an attenuation of cold‐stimulated TG lipolysis by myeloid Sirt6 deficiency (Figure 1f,g). We previously reported that mS6KO mice fed HFD displayed severe insulin resistance (Lee et al., 2017). We, therefore, investigated whether this phenotype of mS6KO is maintained upon cold exposure. While the basal blood glucose levels of both the mS6KO and the WT mice remained the same, the mS6KO mice showed higher glucose levels during the course of the glucose tolerance test (Figure 1h), consistent with the phenotypes of mS6KO under HFD (Lee et al., 2017).

We next examined whether the suppressed heat production in male mS6KO mice was due to the changes in fat beiging/browning response on cold exposure. H&E staining and UCP1 immunostaining revealed that cold (6°C) led to smaller adipocytes with higher UCP1 expression in the scWAT of the WT mice; however, this beiging effect of cold exposure was markedly mitigated in the mS6KO mice (Figure 1i,j). An impairment in the cold‐induced expression of thermogenic markers such as UCP1 and PGC1α in KO‐scWAT was further demonstrated by western blotting and real‐time quantitative RT‐PCR (qPCR) analyses (Figure 1k and Figure S2). Consistent with the absence of any difference between genotypes in wet weight in BAT after cold exposure (Figure 1d), BAT activation remained normal in the mS6KO mice (Figure S3).

We confirmed the overall phenotypes observed in male mS6KO mice in response to cold exposure were recapitulated in female mS6KO mice (Figure S4). In sum, we conclude that myeloid Sirt6 functionally impacts the cold‐stimulated thermogenic response in both male and female mice.

### 2.3 Myeloid‐specific Sirt6 deficiency reduces M2 macrophage content in scWAT upon cold exposure

**FIGURE 2:**
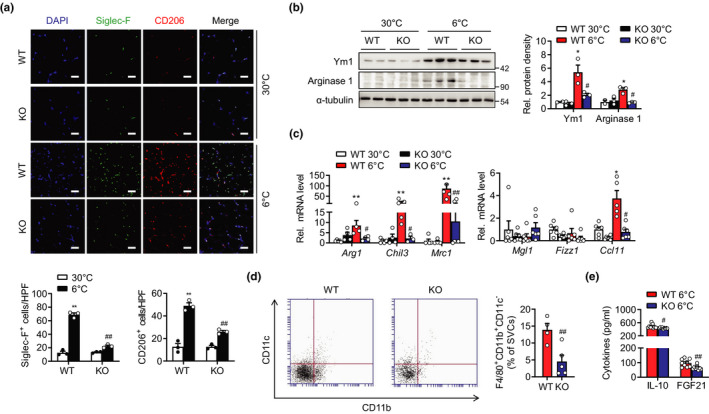
Suppressed recruitment of M2 macrophage in scWAT of male mS6KO mice. mS6KO mice and their WT littermates were housed at 30°C for 1 day and then exposed to cold (6°C) for 3 days. (a) scWAT was immunostained with an antibody against Siglec‐F or CD206. Bars = 25 μm. The numbers of Siglec‐F or CD206‐positive cells were counted in three high‐power fields (HPF) per mouse. The graph shows quantification results (*n* = 3). (b, c) Protein and mRNA levels of M2 markers in scWAT (*n* = 4–6). (d) Stromal vascular cells (SVCs) were prepared from scWAT of the cold‐exposed mice and the composition of M2 macrophage (F4/80^+^CD11b^+^CD11c^−^) was analyzed by flow cytometry. The cell population was expressed as the percentage of SVCs (*n* = 4–5). (e) Serum levels of IL‐10 and FGF21 were measured by ELISA (*n* = 9). Data represent the mean ± SEM. *p < 0.05 and **p < 0.01 vs. WT 30°C; ^#^ *p* < 0.05 and ^##^ *p* < 0.01 vs. WT 6°C

Because myeloid Sirt6 has been found to critically affect the number and type of macrophages in adipose tissue (Lee et al., 2017), we measured macrophage infiltration in scWAT to address how myeloid Sirt6 contributes to browning evoked by cold stress. Immunostaining with anti‐CD206 antibody, western blotting and qPCR analyses for signature genes of M2 macrophages demonstrated that the number of M2 macrophages was elevated in the scWAT of the WT mice upon cold exposure (Figure 2a–c). However, these responses were markedly suppressed in the mS6KO mice. Flow cytometric analysis with SVCs further demonstrated a decreased number of F4/80^+^CD11b^+^CD11c^−^ cells (M2 macrophages) in the scWAT of the mS6KO mice compared with that of the WT mice after cold exposure (Figure 2d). Consistently, the serum level of anti‐inflammatory cytokine IL‐10 and the thermogenic hormone FGF21 were significantly repressed in the mS6KO mice (Figure 2e). These results indicate that upon cold exposure a myeloid Sirt6 deficiency caused a corresponding decrease in M2 macrophage content within scWAT.

**FIGURE 3:**
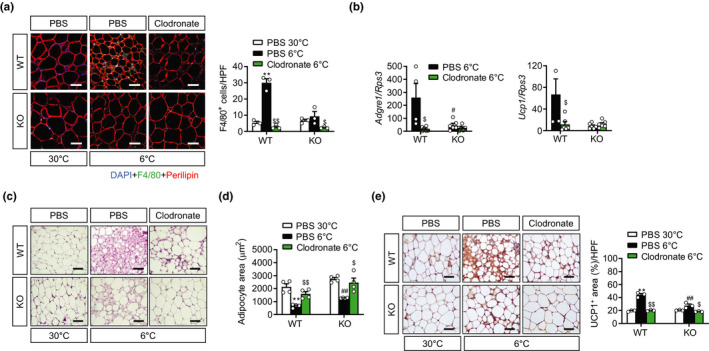
Depletion of macrophages in scWAT by clodronate injection. Eight‐week‐old male mS6KO mice and their WT littermates were subcutaneously injected with liposomes containing either clodronate or PBS for two consecutive days, then housed at 30 or 6°C for a further 3 days. (a) scWAT was stained with H&E or immunostained with an antibody against F4/80 or perilipin. Bars = 25 μm. The number of F4/80‐positive cells were counted in three high‐power fields (HPF) per mouse. Quantification results were from three mice per group. (b) mRNA levels of F4/80 (*Adgre1*) and *Ucp1* in scWAT were determined by qPCR (*n* = 4–6). (c, d) scWAT was stained with H&E and adipocyte cell size was determined (*n* = 4). (e) scWAT was immunostained with a UCP1 antibody. Bars = 25 μm. Quantification results were from three mice per group. Data represent the mean ± SEM. *p < 0.05 and **p < 0.01 vs. PBS 30°C; ^#^ *p* < 0.05 and ^##^ *p* < 0.01 vs. WT PBS 6°C; ^$$^ *p* < 0.01 vs. PBS 6°C

Next, to evaluate the causal relation between M2 macrophages and scWAT browning, we depleted macrophages by subcutaneously injecting the WT and mS6KO mice with a clodronate‐containing liposome suspension, followed by exposure at 6°C for 3 days. Successful depletion of macrophages was verified by F4/80 immunostaining and qPCR analysis for *Adgre1* (encoding F4/80) (Figure 3a,b). Significantly, the infiltration of F4/80‐positive macrophages, the induction of UCP1, and the reduction in adipocyte size by cold exposure were notably abrogated in the scWAT of the WT mice after the clodronate injection, resulting in no difference between the genotypes (Figure 3a‐e).

### 2.4 Myeloid‐specific Sirt6 deficiency reduces eosinophil content in scWAT upon cold exposure

**FIGURE 4:**
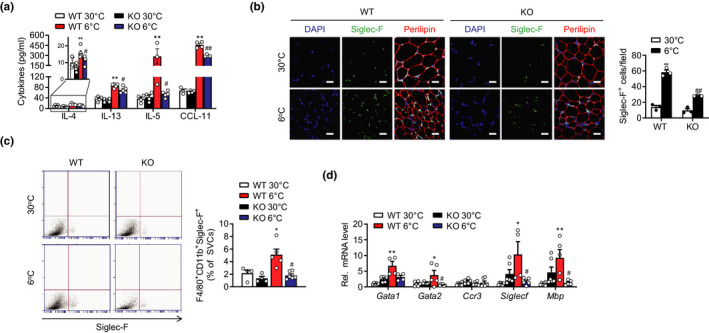
Repressed infiltration of eosinophils in scWAT of mS6KO mice upon cold exposure. mS6KO mice and their WT littermates were housed at 30°C for 1 day and then exposed to cold (6°C) for 3 days. (a) Serum levels of cytokines were analyzed (*n* = 4–6). (b) scWAT was immunostained with an antibody against Siglec‐F or perilipin. Bars = 25 μm. The number of Siglec‐F‐positive cells were counted in three high‐power fields (HPF) per mouse. Quantification results were from three mice per group. (c) Stromal vascular cells (SVCs) were prepared from scWAT and the proportion of eosinophils (F4/80^+^CD11b^+^Siglec‐F^+^) was analyzed by flow cytometry. Each subpopulation was expressed as the percentage of SVCs (*n* = 4–6). (d) qPCR analysis for eosinophil signature genes (*n* = 4–6). Data represent the mean ±SEM. *p < 0.05 and **p < 0.01 vs. WT 30°C; ^#^ *p* < 0.05 and ^##^ *p* < 0.01 vs. WT 6°C

Because IL‐4 together with IL‐13 drives M2 macrophage polarization (Rao et al., 2014) and IL‐5 is a major activator of eosinophils, we quantified these Th2 cytokine levels in the mice. ELISA analysis revealed that all these Th2 cytokines and the eosinophil chemoattractant CCL‐11 were significantly lower in the serum of the mS6KO mice under 6°C (Figure 4a). To identify eosinophils competent for Th2 cytokines production, we immunostained scWAT with Siglec‐F, selectively expressed in mouse eosinophils, after cold exposure for 3 days. Cold exposure increased the number of Siglec‐F‐positive eosinophils in the scWAT of the WT mice, but they were substantially reduced in the mS6KO mice (Figure 4b). This impairment in eosinophil infiltration was also evident by FACS analysis, as the scWAT of the mS6KO mice had fewer F4/80^+^CD11b^+^Siglec‐F^+^ cells (Figure 4c). Accordingly, after cold exposure, the scWAT of mS6KO mice showed lower expression of eosinophil differentiation‐related genes, including *Gata2*, *Siglecf*, and *Mbp* (Figure 4d).

**FIGURE 5:**
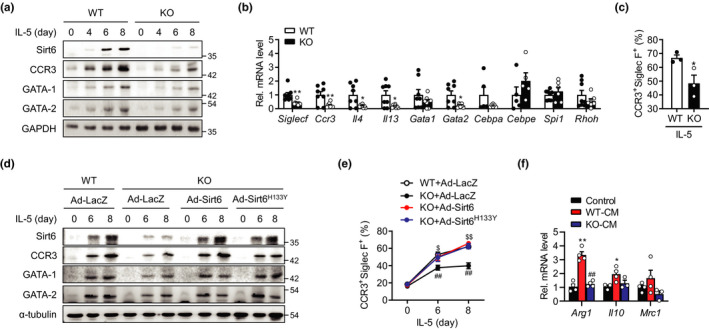
Impairment of eosinophil differentiation in the absence of Sirt6. (a) BMCs from WT or mS6KO mice were treated with 10 ng/ml IL‐5 for indicated time periods following 4 days of ex vivo culture. The protein levels of Sirt6 and eosinophil markers were analyzed by western blotting and quantified (*n* = 3). (b) At day 8 of IL‐5 treatment, the mRNA expression of eosinophil markers and eosinophilic transcription factors was measured by qPCR (*n* = 5–8). (c) BMCs from WT or mS6KO mice were treated with 10 ng/ml IL‐5 for 8 days, and eosinophil differentiation was compared by flow cytometric analysis (*n* = 4–8). (d) Following 3 days of ex vivo culture, BMCs were transduced with Ad‐LacZ, Ad‐Sirt6, and Ad‐Sirt6^H133Y^ and further cultured in the presence of IL‐5 for the indicated time points. The protein levels of the eosinophil markers were analyzed by western blotting and quantified (*n* = 3). (e) After IL‐5 treatment, FACS analysis was performed at the indicated time points (*n* = 3). (f) Naïve bone marrow macrophages were incubated for 8 h with conditioned media (CM) collected between day 7 and day 8 from ex vivo eosinophil cultures of WT or mS6KO. mRNA for M2 macrophage genes were also analyzed (*n* = 3–4). Data represent the mean ±SEM. *p < 0.05 and **p < 0.01 vs. WT or Control; ^#^ *p* < 0.05 and ^##^ *p* < 0.01 vs. WT+Ad‐LacZ or WT‐CM; ^$^ *p* < 0.05 and ^$$^ *p* < 0.01 vs. KO+Ad‐LacZ

Eosinophils differentiate from myeloid precursors (Rothenberg & Hogan, 2006). To explain the reduced number of eosinophils in the scWAT of the mS6KO mice, we explored whether myeloid Sirt6 deficiency could directly suppress eosinophil differentiation. Bone marrow cells (BMCs) from WT or mS6KO mice were isolated and induced to differentiate into eosinophils pursuant to a well‐established cytokine regimen (Dyer et al., 2008) in which SCF and FLT‐3 support progenitor growth, whereas IL‐5 induces the terminal differentiation of committed progenitors. We found that IL‐5 treatment resulted in a gradual increase in Sirt6 protein levels over 8 days of differentiation in WT mice (Figure 5a). Eosinophil marker gene CCR3 (a specific receptor for CCL‐11) and major transcription factors of eosinophil differentiation GATA‐1 and GATA‐2 (Yu et al., 2002) were significantly induced during the same period, suggesting maximum eosinophil expansion (Dyer et al., 2008). However, eosinophil cultures from mS6KO exhibited noticeably lower protein and mRNA levels of CCR3, GATA‐1, GATA‐2, and other eosinophil transcripts, such as *Siglecf*, *Il4*, and *Il13* (Figure 5a,b, and Figure S5a). CEBPα *(Cepba)* mRNA, which is involved in eosinophil lineage commitment and maturation, was also significantly down‐regulated in mS6KO (Figure 5b). Flow cytometric analysis at day 8 after IL‐5 treatment confirmed the marked reduction of CCR3^+^Siglec‐F^+^ eosinophils in mS6KO (Figure 5c and Figure S5b). In sum, we concluded that myeloid Sirt6 deficiency partially but significantly mitigated eosinophil differentiation.

Next, to assess whether restoration of Sirt6 is sufficient to rebuild their ability to differentiate into eosinophils, we re‐expressed Sirt6 into BMCs from mS6KO. The number of repressed CCR3^+^Siglec‐F^+^ eosinophils and eosinophil marker genes in the mS6KO mice were indeed restored by Ad‐Sirt6 infection (Figure 5d,e and Figure S5c,d). Interestingly, adenovirus infection with deacetylase‐mutant Sirt6 (Ad‐Sirt6^H133Y^) produced similar results with wild Sirt6, indicating that the deacetylase activity of Sirt6 is dispensable for regulating eosinophil differentiation.

Because cold‐induced M2 macrophage infiltration into scWAT was suppressed in mS6KO than in WT (Figure 2), we next analyzed whether myeloid Sirt6 deficiency directly affected the ability of eosinophil to regulate M2 macrophage polarization. When naïve bone marrow macrophages were incubated with conditioned media (CM) collected from eosinophil cultures from WT or mS6KO mice, mRNA levels of M2 macrophage marker genes such as *Arg1* and *Il10* were significantly increased by CM from WT eosinophils but not by CM from mS6KO eosinophils (compared with control media) (Figure 5f), evincing a controlling role for eosinophil Sirt6 in M2 macrophage polarization.

### 2.5 Cooperative binding of Sirt6 and p300 to GATA‐1 enhances its transcriptional activity

**FIGURE 6:**
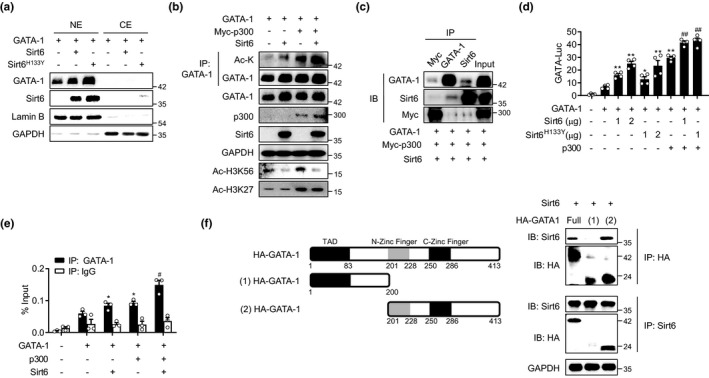
Enhancement of GATA‐1 transcriptional activity by Sirt6 or Sirt6^H133Y^. (a) HEK293T cells were transfected with plasmid encoding GATA‐1, Sirt6, and deacetylase mutated Sirt6 (Sirt6^H133Y^) as indicated. GATA‐1 protein levels in the nuclear extract (NE) and cytoplasmic extract (CE) were measured. (b) HEK293T cells were transfected with indicated plasmids and total cell lysates were used for co‐IP experiments. Histone H3 acetylation at lysine (K) are indicative of Sirt6 or p300 overexpression. (c) After transfection in HEK293T cells as indicated, IP followed by immunoblotting were performed with specific antibodies. (d) The relative GATA‐1 luciferase activity in the cell lysates was measured (*n* = 4). (e) ChIP was performed using anti‐GATA‐1 antibody and IgG. Enrichment of GATA‐1 on the *Ccr3* promoter region after over expression of Sirt6 and/orp300 was analyzed by qPCR. (f) Binding of Sirt6 to full length or truncated GATA‐1 was analyzed by co‐IP. Values are the mean ± SEM. *p < 0.05 and **p < 0.01 vs GATA‐1; ^#^ *p* < 0.05 and ^##^ *p* < 0.01 vs. GATA‐1+p300

On the basis of our determination that the mS6KO mice exhibited reduced GATA‐1 and GATA‐2 protein levels in eosinophils (Figure 5a,b), and that Sirt6 interacted with and increased the protein stability of GATA‐1 with no effect on GATA‐2 (Figure S6a–d), we concentrated on the GATA‐1 transcription factor for further analysis. In order to correlate the subcellular localization of GATA‐1 and its activity, we first measured GATA‐1 protein levels in nuclear and cytosolic fractions after Sirt6 overexpression. Transfection of HEK293T cells with either WT or mutant forms of Sirt6 (deacetylase mutant H133Y or deacetylase and ADP‐ribosyltransferase mutant S56Y) increased nuclear levels of GATA‐1 (Figure 6a and Figure S7a), being consistent with GATA‐1 protein stabilization (Figure S6c). Because the transcriptional activity of GATA‐1 was reported to be dependent on the acetylation which is mediated by CBP/p300 acetyltransferase (Boyes et al., 1998; Hung et al., 1999; Lamonica et al., 2006), we addressed whether GATA‐1 acetylation was altered by Sirt6. GATA‐1 acetylation was slightly increased by Sirt6 and further enhanced by p300 co‐transfection (Figure 6b), coincident with the physical interaction among Sirt6, p300, and GATA‐1 as demonstrated by Co‐IP analysis (Figure 6c). Interestingly, both Sirt6^H133Y^ and Sirt6^S56Y^ also increased the basal‐ and p300‐mediated acetylation of GATA‐1 (Figure S7b). More importantly, GATA‐1 transcriptional activity, as measured by GATA‐luciferase reporter gene analysis, was increased by co‐transfection with Sirt6, Sirt6^H133Y^, or Sirt6^S56Y^, and further enhanced by p300 (Figure 6d and Figure S7c). Physical interaction among GATA‐1, Sirt6, and p300, and the enhancement of GATA‐1 transcriptional activity by Sirt6, were also confirmed in a human eosinophil cell line AML14.3D10 (Figure S7d,e).

Finally, ChIP was performed to test GATA‐1 enrichment on the *Ccr3* promoter using an anti‐GATA‐1 antibody in HEK293 cells with p300 or Sirt6 overexpression. The results showed that GATA‐1 enrichment at the *Ccr3*’s promoter region was significantly augmented when transfected with either p300 or Sirt6, and further enhanced by co‐transfection of p300 and Sirt6 (Figure 6e). We lastly found that Sirt6 interacted with zinc‐finger containing region of GATA‐1 (Figure 6f), which was coherent with the previous reports indicating zinc‐finger region of GATA‐1 interacts with other proteins such as Cdk9 or deubiquitylase USP7 (Bottardi et al., 2013; Liang et al., 2019). When viewed together, these results indicate that Sirt6 and p300 directly bind to GATA‐1 and cooperate in regulating GATA‐1 target gene expression.

## 3 DISCUSSION

Previous studies have shown that eosinophils accumulate in adipose tissues in response to body weight loss, cold exposure, and helminth infection, protecting subjects against obesity and insulin resistance (Hams et al., 2013; Molofsky et al., 2013; van den Berg et al., 2017; Wu et al., 2011). Obesity, in contrast, suppresses adipose eosinophil accumulation. These processes are tightly linked to type 2 immune responses; eosinophils secrete cytokines to stimulate M2‐like macrophages in adipose tissue, which triggers expression of UCP1 and adipose tissue thermogenesis (Wu et al., 2011). In this study, we demonstrate that myeloid Sirt6 is an important regulator of eosinophil development, and consequently WAT browning and thermogenic adaptation to cold. It is noteworthy that aP2‐Cre‐mediated Sirt6 knockout mice, in which Sirt6 could be deleted in myeloid cells as well as in adipocytes (Fu et al., 2002; Makowski et al., 2001), exhibited an impaired UCP1 expression in response to metabolic stress (Xiong et al., 2017). Our results establish a role for myeloid Sirt6 in the regulation of eosinophil differentiation and the development of beige fat.

It is notable that BAT, unlike scWAT, was not impaired in adaptive thermogenesis in the mS6KO mice, indicating that myeloid Sirt6 activity is not a prerequisite for BAT activation. What accounts for this difference between BAT and scWAT in the mS6KO mice? An explanation remains elusive. One possible explanation is that BAT is highly efficient and active in heat generation due to its constitutively high expression of UCP1 relative to scWAT in mice. Alternatively, BAT may be relatively resistant to immune cell infiltration upon metabolic stress (Fitzgibbons et al., 2011).

Our observations of the reductions in circulating Th2 cytokines and scWAT M2 macrophages in mS6KO mice support the view that M2 macrophages are the primary actors in beige fat development. Previously, we have demonstrated that Sirt6 deletion from myeloid cells reduced alternative activation of macrophages to M2‐like cells, and that this was mediated by the attenuated IL‐4/Akt signaling (Koo et al., 2019). In this study, we demonstrated macrophage‐depletion using clodronate‐ameliorated adaptive thermogenesis on cold exposure in both WT and mS6KO mice, confirming that M2 macrophages are an endogenous regulator that orchestrates this circuit. These results are consistent with the work of other researchers, who have shown that deleting insulin receptor substrate 2 in LysM‐expressing cells increases IL‐4/Akt signaling in bone marrow‐derived macrophages, with concomitant upregulation of WAT browning in mice (Rached et al., 2019).

Several studies have reported that disruption of the eosinophil‐M2 macrophage axis limits beige cell biogenesis (Huang et al., 2017; Qiu et al., 2014; Rao et al., 2014). Consistent with these reports, we demonstrate that myeloid Sirt6 deficiency affects both eosinophils and M2 macrophages in scWAT of mS6KO mice subjected to cold, confirming a close link between eosinophils and M2 macrophages. How do eosinophils stimulate M2 macrophage recruitment in scWAT and promote WAT browning? Eosinophils secrete Th2 cytokine IL‐4/IL‐13, which promotes M2 macrophage polarization (Wu et al., 2011), which in turn stimulates AT browning by a mechanism that has not yet been identified. Although early research suggested that norepinephrine secreted from M2 macrophages was responsible for AT browning/thermogenesis (Nguyen et al., 2011), this was disconfirmed by subsequent studies (Fischer et al., 2017; Pirzgalska et al., 2017). One recent study reported that eosinophils may directly drive beiging activation in adipocytes through paracrine signaling, that is, secreting eosinokines such as meteorin‐like or IL‐33 (Knights et al., 2020). Our study supports, at least in part, the existence of an eosinophil‐Th2 cytokines‐M2 macrophage circuit in WAT browning. Specifically, our experiments showed that mS6KO mice displayed lower serum levels of CCL‐11 and Th2 cytokines with reduced accumulation of eosinophils and M2 macrophages in scWAT, and that Sirt6 deficiency could inhibit the differentiation of eosinophils from bone marrow precursors.

Eosinophil differentiation occurs through a complex series of events involving various transcription factors, including GATA members. While it was well‐known that GATA family transcriptional activity varied according to acetylation status (Huo & Zhang, 2005), whether GATA‐1 is (de)acetylated pursuant to eosinophil differentiation had, until now, not been explored. In this study, we found that Sirt6 recruited p300 and GATA‐1 to promote GATA‐1 acetylation and transcriptional activation of its target genes. Intriguingly, Sirt6’s enzymatic activity as a deacetylase or ADP‐ribosyltransferase was not required for GATA‐1 protein stabilization, its acetylation, chromatin occupancy, transcriptional activity, or eosinophil differentiation. A similar finding was also observed in a recent study by Peng et al. (2020) in which Sirt6 was found to have regulated GATA‐4 transcriptional activity independent of its deacetylase activity but to act as a scaffold protein recruiting an acetyltransferases TIP60. It is noteworthy that Sirt6 has a very weak deacetylase activity such that its catalytic efficiency is about 100–1000 times lower than the most active sirtuins (Pan et al., 2011), in agreement with its role as a cofactor protein.

To date, two groups have reported the acetylation residues of GATA‐1 which was mediated by CBP/p300 acetyltransferase; that is, K246, K252, K312 in murine GATA‐1 (Hung et al., 1999) and the corresponding sites in chicken GATA‐1 (Boyes et al., 1998). They both concluded that GATA‐1 acetylation is required for transcriptional activity. However, Hung et al. (1999), suggested GATA‐1 acetylation is not important in its DNA binding, as opposed to Boyes et al. (1998), but that acetylation of GATA‐1 increases its binding affinity to CBP and so recruits CBP, leading to acetylation of both histone and GATA‐1. Taking these reports and our current results into consideration, we suggest that Sirt6 regulates GATA‐1 function through multiple mechanisms such as acetylation, DNA binding, and protein stability. Co‐IP study showed that Sirt6 directly interacts with GATA‐1 zinc‐finger region. Further studies of ChIP and co‐IP showed that both DNA binding and acetylation of GATA‐1 were increased by Sirt6 and p300 alone and even more increased by their co‐presence. Accordingly, GATA‐1 transcription activity was increased by Sirt6 and/or p300, as demonstrated by GATA‐1 reporter gene analysis. Finally, GATA‐1 activation by Sirt6 resulted in the induction of the eosinophil target gene. Taken together, our data establish the Sirt6, independent of deacetylase or ADP‐ribosyltransferase, as a novel‐positive regulator of GATA‐1 function capable of modulating eosinophil differentiation. Further investigation will be required to identify the acetylation sites in GATA‐1 and its functionality regulating eosinophil differentiation.

In summary, we have demonstrated that myeloid Sirt6 acts as a GATA‐1 transcriptional cofactor. As GATA‐1 is critical for eosinophil differentiation, the conclusion of this study may be summarized as follows: myeloid Sirt6 → eosinophil differentiation and infiltration into scWAT → Th2 cytokine production → M2 macrophage polarization → UCP1 expression in adipocytes → WAT browning and heat generation. It is through this process that myeloid Sirt6 may counteract metabolic dysregulation and insulin resistance. When read in conjunction with our recent observations of an inverse correlation between Sirt6 protein levels and metabolic and inflammatory diseases in humans (Moon et al., 2019; Song et al., 2019; Woo et al., 2018), this study suggests a potential role for myeloid Sirt6 in human fat beiging.

## 4 EXPERIMENTAL PROCEDURES

### 4.1 Animal experiments

mS6KO mice (*Sirt6^fl^* ^/^ *^fl^*:*LysM*) were generated by crossing *Sirt6^fl^* ^/^ *^fl^* mice (B6;129‐*Sirt6* ^tm1Ygu^/J) and *LysM*‐*Cre* mice (B6.129P2‐*lyz2* ^tm1(cre)Ifo^/J) as previously described (Lee et al., 2017). Mice were housed in a controlled barrier facility (12 h light/dark cycle, 23 ± 1°C, 60%–70% humidity) and fed ad libitum with standard chow. For cold challenge experiments, 8‐week‐old male or female WT and mS6KO mice were housed at 6 or 30°C for 3 days after acclimatization at thermoneutrality (30°C) for one day. Body surface temperature surrounding inguinal scWAT and interscapular BAT was determined by infrared thermography with an FLIR T440 infrared camera (Seoul, Korea). For the glucose tolerance test, 16 h‐fasted mice received a glucose (1 g/kg) intraperitoneally and the glucose concentration was evaluated in tail‐tip blood at the indicated time points. To deplete macrophages in scWAT, 100 μl clodronate liposomes (LIPOSOMA BV, Amsterdam, Netherland) were administered once daily for 4 days into inguinal WAT on one side, and an identical amount of control (PBS) liposomes was administered on the other side. This study protocol was approved by the Institutional Animal Care and Use Committee of Chonbuk National University (Permit No: CBNU 2017‐0091).

### 4.2 Histology

Histological sections (6 μm) were cut from formalin‐fixed paraffin‐embedded tissue blocks and were stained with hematoxylin–eosin (H&E) under standard conditions. Immunohistochemical staining was performed using the DAKO Envision system (DAKO, Carpinteria, CA, USA). Sections were immunostained with antibodies against UCP1 (Sigma‐Aldrich, St Louis, MO, USA). Peroxidase activity was detected with 3‐amino‐9‐ethyl carbazole. The adipocyte area in selected fat tissue sections was measured using iSolution DT 36 software (Carl Zeiss, Oberkochen, Germany).

For immunofluorescence staining, frozen sections were stained with anti‐Siglec‐F (Abcam, Cambridge, UK) or anti‐CD206 (Abcam) antibodies at 4°C overnight. Immunofluorescence analyses were performed to identify macrophages and eosinophils by staining tissues with a combination of anti‐perilipin (Fitzgerald, MA, USA) with either anti‐F4/80 or anti‐Siglec‐F antibodies, respectively. After incubation with the corresponding fluorochrome‐conjugated secondary antibodies, the sections were mounted and visualized using an LSM510 confocal laser scanning microscope (Carl Zeiss) installed in the Center for University‐Wide Research Facilities (CURF) at Chonbuk National University.

### 4.3 Fluorescence‐activated cell sorting (FACS) analysis

Adipose tissue SVCs and bone marrow‐derived eosinophils were incubated in FACS buffer containing 2% FBS with Fc Block (BD Biosciences, San Jose, CA, USA) for 30 min at 4°C prior to staining with following antibodies (all from BD Biosciences): F4/80 (1 μg/ml), CD11b (0.4 μg/ml), CD11c (0.4 μg/ml), Siglec‐F (0.4 μg/ml), or CCR3 (0.4 μg/ml) for 30 min at 4°C. Stained cells were gently washed and re‐suspended in FACS buffer, then analyzed using a FACS Calibrator instrument (BD Biosciences). Unstained, single‐stained, and fluorescence‐minus‐one controls were used for setting compensation and gates.

### 4.4 Ex vivo eosinophil differentiation

Eosinophils were generated from BMCs as described previously (Dyer et al., 2008). In brief, BMCs (1 × 10^6^/ml) were cultured in Iscove's modified Dulbecco's medium containing 20% FBS, 100 IU/ml penicillin and 10 μg/ml streptomycin, 2 mM glutamine and 1x non‐essential amino acids and 1 mM sodium pyruvate and 50 μM β‐mercaptoethanol supplemented with 100 ng/ml recombinant murine stem‐cell factor (rmSCF, PeproTech, RockyHill, NJ, USA) and 100 ng/ml fms‐like tyrosine kinase 3 ligand (rmFLT3L, PeproTech) from day 0 to day 4. On day 4, the medium was replaced with a medium containing 10 ng/ml rmIL‐5 (R&D Systems, Minneapolis, MN, USA) only and was changed with 10 ng/ml rmIL‐5 every other day until day 12.

To carry out co‐culture of bone marrow macrophages with eosinophil‐CM, CM from day 7 to day 8 (24 h) of ex vivo eosinophil cultures derived from WT or mS6KO were collected. BMCs from C57BL/6 mice were incubated with M‐CSF for 48 h followed by 8 h‐incubation with CM, and mRNA of M2 macrophage genes was analyzed.

### 4.5 Co‐immunoprecipitation (Co‐IP) and western blotting

Tissues and cells were homogenized in Protein Extraction Reagent (Thermo Fisher Scientific, Waltham, MA, USA). Homogenates (20 μg of total protein) were separated by SDS‐PAGE and transferred to nitrocellulose membranes. Blots were probed with primary antibodies against Sirt6, GATA‐1, Ac‐K, Ac‐H3K27 (Cell Signaling, Beverly, MA, USA), PGC1α, PRDM16, CCR3, IL‐4Rα (Abcam), α‐tubulin, UCP1 (Sigma‐Aldrich), myc, HA tag (Thermo Fisher Scientific, Waltham, MA, USA), Ac‐H3K27 (Active Motif, Carlsbad, CA, USA), Ym1 (Stemcell Technologies, Cologne, Germany), and arginase 1 (Arg1, Santa Cruz Biotechnology, Dallas, TX, USA). Immunoreactive bands were detected with a Las‐4000 imager (GE Healthcare Life Science, Pittsburgh, PA, USA).

### 4.6 Cell culture and transient transfection

HEK293T obtained from the American Type Culture Collection (Manassas, VA, USA) were mock‐transfected or transfected with 0.5–2 μg of GATA‐1, Sirt6, Sirt6^H133Y^, Sirt6^S56Y^, and myc‐p300 using Lipofectamine 3000 (Invitrogen, Carlsbad, CA, USA). AML14.3D10 is a standard human myeloid leukemic cell line model used for eosinophil studies (Baumann & Paul, 1998). The cell line we used was a gift from Professor YunJae Jung (Gachon University, Incheon, Korea). This cell line was grown in a humidified atmosphere at 37°C (5% CO_2_) in RPMI‐1640 media (Invitrogen) supplemented with 10% FBS, 2 mM glutamine, 100 units/ml of penicillin, and 100 μg/ml streptomycin. On day 1 of differentiation, cells were cultured at a cell density of 2 × 10^5^ cells/ml and supplemented with 0.4 mM butyric acid. On day 4, the cells were diluted to 2 × 10^5^ cells/ml and the concentration of butyric acid was adjusted to 0.4 mM. The cells on day 7 were used for Co‐IP and GATA‐1 reporter gene assay.

For the GATA‐1 reporter gene assay, 1 μg of GATA promoter luciferase (Qiagen, Hilden, Germany) was transfected using lipofectamine (HEK293T) or by electroporation (AML14.3D10). After 48 h, cells were harvested in reporter lysis buffer (Promega, Madison, WI, USA). Luciferase activity was measured using a Dual‐Luciferase Reporter Assay (Promega) by Lumat LB 9507 (Berthold, Bad Wildbad, Germany).

### 4.7 RNA isolation and qPCR

Total RNA was extracted from frozen liver tissue using an RNA Iso kit (TaKaRa, Tokyo, Japan). First‐strand cDNA was generated using the random hexamer primer provided in the first‐strand cDNA synthesis kit (Applied Biosystems, Foster City, CA, USA). Specific primers for each gene (Table S1) were designed using PrimerBank (https://pga.mgh.harvard.edu/primerbank). qPCR reactions were performed under standard conditions using an ABI QuantStudio6 Flex Real‐time PCR System (Applied Biosystems).

### 4.8 ChIP assay

HEK293T cells were cross‐linked by incubating cells in 1% formaldehyde for 10 min at room temperature. Cross‐linking was stopped by 5 min of incubation with 125 mM glycine. Cells were lysed with cytosolic lysis buffer. After centrifugation, the following processes were performed with Simple Chip Enzymatic Chromatin IP Kits (Cell Signaling Technology, Danvers, MA, USA). Each sample was immunoprecipitated with 2 μg of anti‐GATA‐1 or non‐specific IgG (Cell Signaling Technology). The obtained DNA was analyzed by qPCR with *Ccr3* promoter primer (forward: 5′‐TTTACTAATGTCTGTGATCTGATGG‐3′; reverse: 5′‐TGGAAAAGCGACACCTACCT‐3′).

### 4.9 Statistical analysis

Data are expressed as the mean ±standard error of the mean (SEM). Statistical comparisons were performed using one‐way analysis of variance followed by Fisher's post hoc analysis. The significance of differences between the two groups was determined using Student's unpaired *t*‐test using GraphPad Prism, version 9.0.0 (San Diego, CA, USA). A *p*‐value of less than 0.05 was considered significant.

## CONFLICT OF INTEREST

The authors declare that they have no conflict of interest.

## AUTHORS’ CONTRIBUTIONS

IHB, DP, YL, and HC performed the experiments and analyzed the data. BHP and EJB designed the experiments, interpreted the data, and wrote the manuscript. All authors reviewed the manuscript.

## Supporting information

Supplementary Material

## Data Availability

The datasets generated during and/or analyzed during this study are available from the corresponding author on reasonable request.
