# Supplementary material for: Sirtuin 6 promotes eosinophil differentiation by activating GATA‐1 transcription factor
Source: Aging Cell. 2021 Jun 14;20(7):e13418. doi: 10.1111/acel.13418 (PMC8282249; doi:10.1111/acel.13418)
Supplement: Supplementary file 1 — Supplementary Material [file ACEL-20-e13418-s001.docx]

**­­Sirtuin 6 promotes eosinophil differentiation by activating GATA-1 transcription factor**

In Hyuk Bang^1^, **Dami Park^1^**, Youngyi Lee^1^, Hwangeui Cho^2^, Byung-Hyun Park^1¶^, and Eun Ju Bae^2¶^

^1^Department of Biochemistry and Molecular Biology, Chonbuk National University Medical School, Jeonju, Jeonbuk 54896, Republic of Korea

^2^College of Pharmacy, Chonbuk National University, Jeonju, Jeonbuk 54896, Republic of Korea

Contents

1. Supplementary table
2. Supplementary figures

**Table S1. Sequences and accession numbers for primers (forward, FOR; reverse, REV)**

| Gene | Sequences for primers | Accession No. |
| --- | --- | --- |
| *Arg1* | FOR: CAGAAGAATGGAAGAGTCAG | NM_007482 |
|  | REV: CAGATATGCAGGGAGTCACC |  |
| *Chil3* | FOR: GGGCATACCTTTATCCTGAG | NM_009892 |
|  | REV: CCACTGAAGTCATCCATGTC |  |
| *Adgre1* | FOR: TTTCCTCGCCTGCTTCTTC | NM_010130 |
|  | REV: CCCCGTCTCTGTATTCAACC |  |
| *Gata1* | FOR: TCTCATCCGGCCCAAGAAGCG | NM_008089 |
|  | REV: GTGGGCGGTTCACCTGATGG |  |
| *Gata2* | FOR: TCTGGCGACGAGATGGCACG | NM_008090 |
|  | REV: TTTGCACAACAGGTGCCCGC |  |
| *Siglecf* | FOR: CTGGCTACGGACGGTTATTCG | NM_145581 |
|  | REV: GGAATTGGGGTACTGGACTTG |  |
| *Ccr3* | FOR: CATAGGGTGTGGTCTCAAAGC | NM_009914 |
|  | REV: AAAGGACTTAGCAAAATTCACCA |  |
| *Il13* | FOR: TCGGCATTTTGAACGAGGTC | NM_008355 |
|  | REV: GAAAAGCCCGAAAGAGTCTC |  |
| *Mbp* | FOR: GCAAACGCTTTCGATGGGTTG | NM_008920 |
|  | REV: ACACAGTGAGATAGACGCCAG |  |
| *Ucp1* | FOR: CAAAAACAGAAGGATTGCCGAAA | NM_009463 |
|  | REV: TCTTGGACTGAGTCGTAGAGG |  |
| *Dio2* | FOR: CAGCTTCCTCCTAGATGCCTA | NM_010050 |
|  | REV: CTGATTCAGGATTGGAGACGTG |  |
| *Ucp3* | FOR: CCGATTTCAAGCCATGATACGC | NM_009464 |
|  | REV: CCTGGCGATGGTTCTGTAGG |  |
| *Prdm16* | FOR: TGCTGACGGATACAGAGGTGT | NM_027504 |
|  | REV: CCACGCAGAACTTCTCGCTAC |  |
| *Ppargc1a* | FOR: TATGGAGTGACATAGAGTGTGCT | NM_008904 |
|  | REV: GTCGCTACACCACTTCAATCC |  |
| *Elovl3* | FOR: TTCTCACGCGGGTTAAAAATGG | NM_007703 |
|  | REV: GGCCAACAACGATGAGCAAC |  |
| *Elovl6* | FOR: GAAAAGCAGTTCAACGAGAACG | NM_130450 |
|  | REV: AGATGCCGACCACCAAAGATA |  |
| *Mrc1* | FOR: CTCTGTTCAGCTATTGGACGC | NM_008625 |
|  | REV: TGGCACTCCCAAACATAATTTGA |  |
| *Mgl1* | FOR: CAATGTGGTTAGTTGGATCGGC | NM_001204252 |
|  | REV: CCCAGTTCTTAAAGCCTTTCTCA |  |
| *Fizz1* | FOR: CCAATCCAGCTAACTATCCCTCC | NM_020509 |
|  | REV: ACCCAGTAGCAGTCATCCCA |  |
| *Ccl11* | FOR: GAATCACCAACAACAGATGCAC | NM_011330.3 |
|  | REV: ATCCTGGACCCACTTCTTCTT |  |
| *Il4* | FOR: GGTCTCAACCCCCAGCTAGT | NM_0210283 |
|  | REV: GCCGATGATCTCTCTCAAGTGAT |  |
| *Cebpa* | FOR: CAAGAACAGCAACGAGTACCG | NM_007678 |
|  | REV: GTCACTGGTCAACTCCAGCAC |  |
| *Cebpe* | FOR: GCAGCCACTTGAGTTCTCAGG | NM_207131 |
|  | REV: GATGTAGGCGGAGAGGTCGAT |  |
| *Spi1* | FOR: TTACAGGCGTGCAAAATGGAA | NM_011355 |
|  | REV: GACGTTGGTATAGCTCTGAATCG |  |
| *Rhoh* | FOR: AACCCACGGTGTACGAGAATA | NM_001081105 |
|  | REV: GGGCCGGATACTTCTGAAGG |  |
| *Sirt6* | FOR: CTCCAGCGTGGTTTTCCACA | NM_181586 |
|  | REV: GCCCATGCGTTCTAGCTGA |  |
| *Rps3* | FOR: GGCAGTGTGACAGGTTGAAG | NM_008084 |
|  | REV: ATCAGAGAGTTGACCGCAGTTG |  |

**
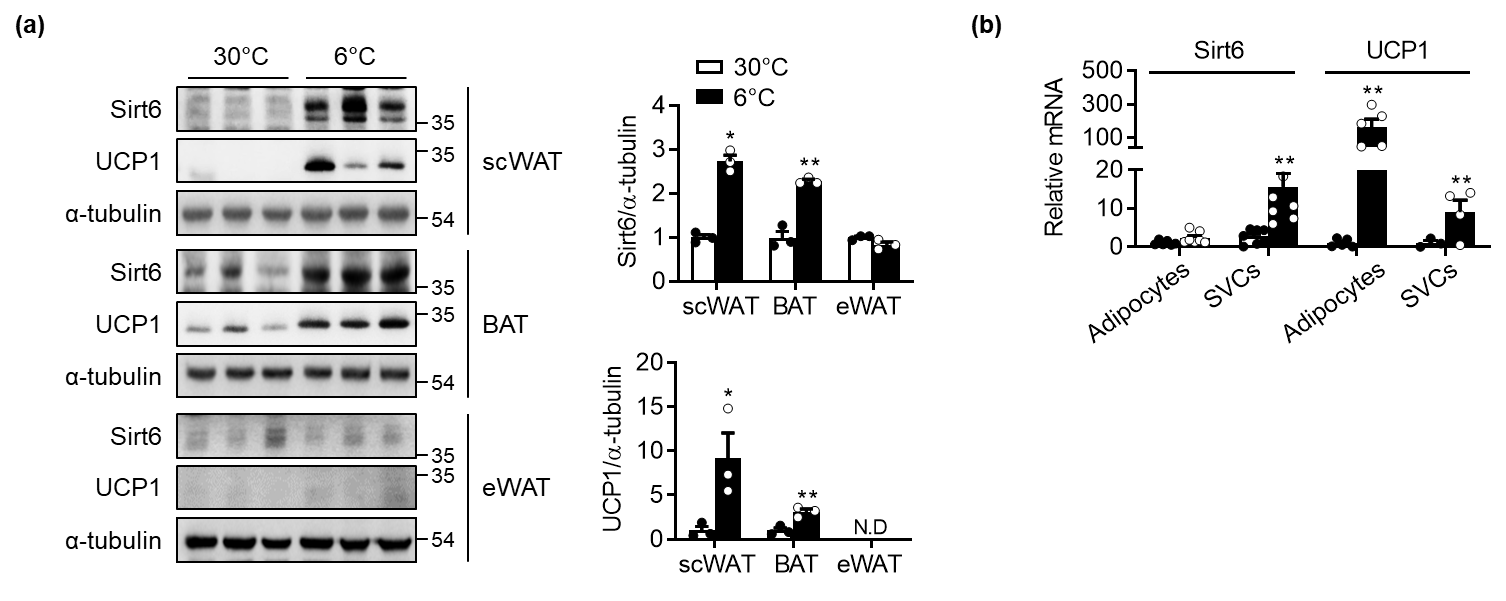
**

**Figure S1. Induction of Sirt6 in adipose tissues after cold exposure.** (a) Eight-week-old male C57BL/6J mice were housed at 30°C for 1 day before cold exposure at 6°C for 3 days. The protein levels of Sirt6 and UCP1 in adipose tissues were analyzed by Western blotting. The relative protein levels of Sirt6 and UCP1 were determined (n=3). (b) Stromal vascular cells (SVCs) and mature adipocytes were isolated from scWAT of C57BL/6J mice after housing at 30°C or 6°C for 3 days. The relative mRNA levels of Sirt6 and UCP1 were determined (n=3-5). Data represent the mean ± SEM. ^*^*p*<0.05 and ^**^*p*<0.01 vs. 30°C. eWAT, epididymal white adipose tissue; scWAT, subcutaneous white adipose tissue, BAT, brown adipose tissue.


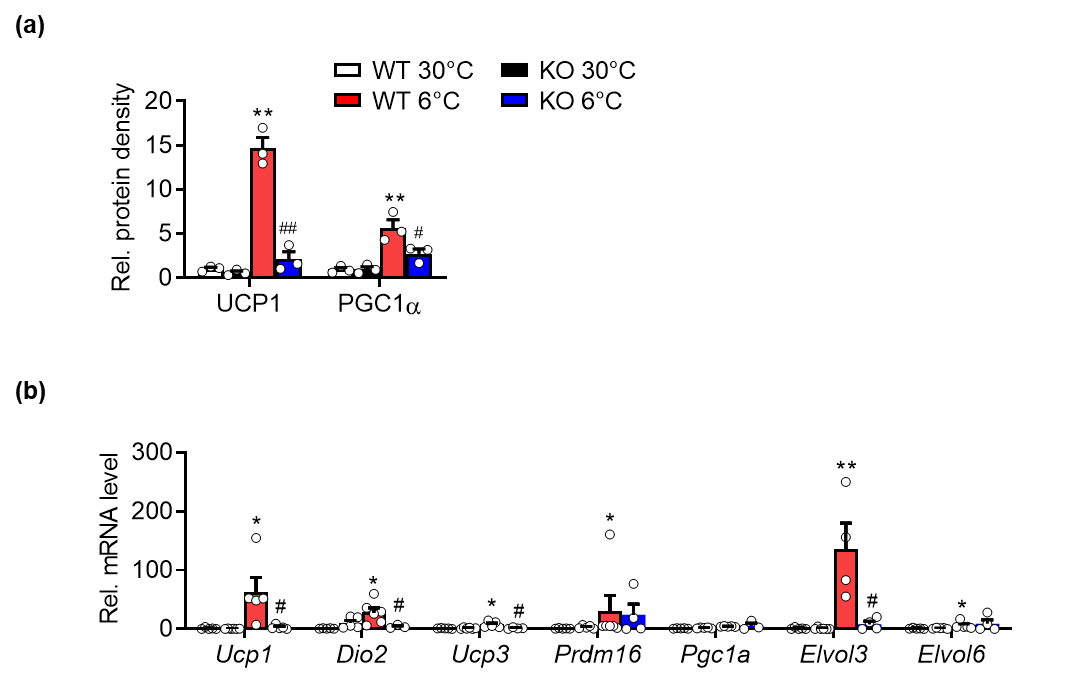


**Figure S2. Impaired beiging in male mS6KO mice.** (a) The band intensities shown in Figure 1k were quantified by densitometry (n=3). The band intensity of each protein was normalized to the intensity of the α-tubulin band. (b) mRNA levels of thermogenic genes in scWAT were determined by qPCR (n=5-6). Data represent the mean ± SEM. ^*^*p*<0.05 and ^**^*p*<0.01 vs. WT 30°C; ^#^*p*<0.05 and ^##^*p*<0.01 vs. WT 6°C.


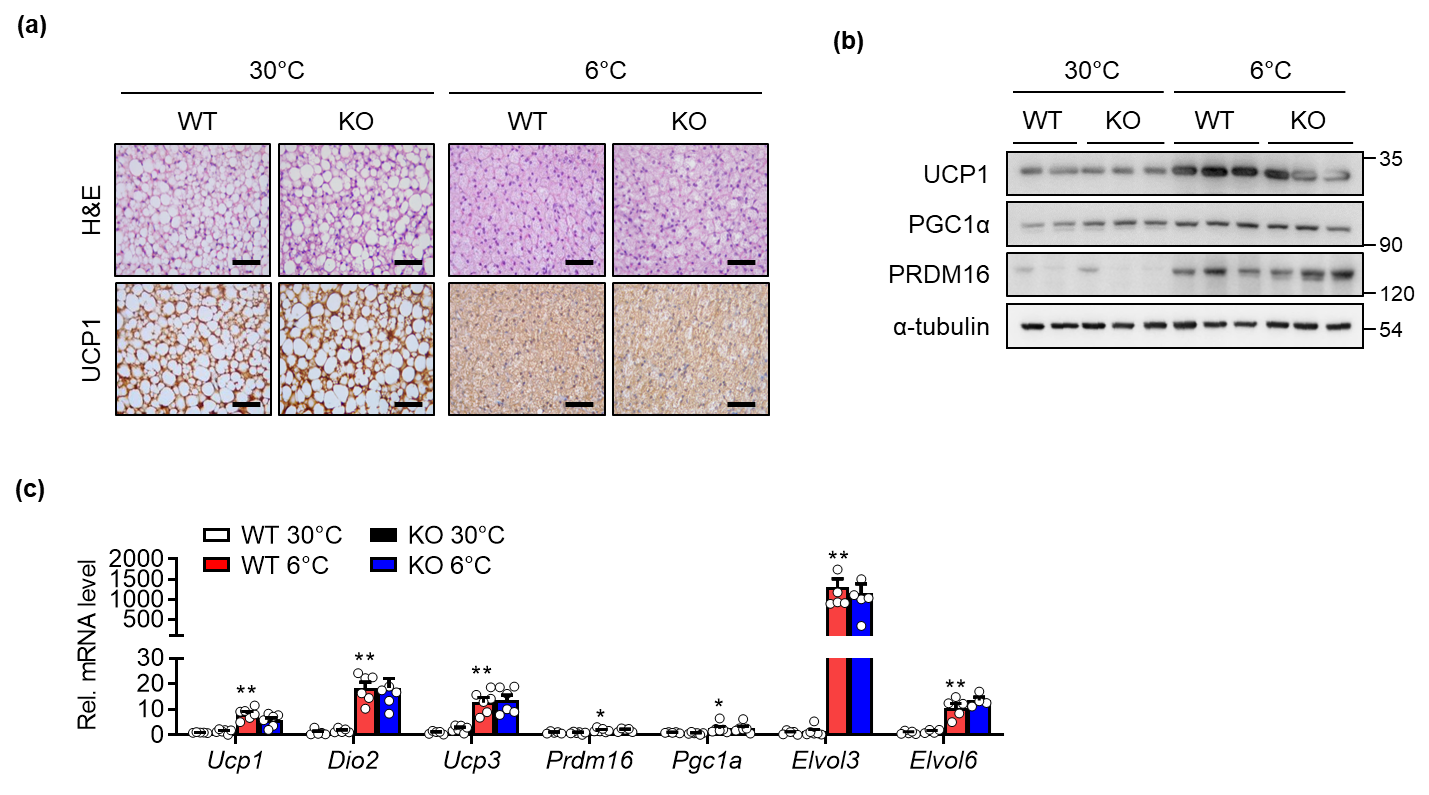


**Figure S3. No change by mSirt6 deficiency in BAT activation following cold exposure.** All experimental procedures are the same as described in Figure 2. (a) Representative H&E-stained and UCP1-immunostained sections from WT or mS6KO mice. Bars=25 μm. (b, c) Protein and mRNA levels of thermogenic markers were determined by Western blotting and qPCR, respectively (n=5-6). Data represent the mean ± SEM. ^*^*p*<0.05 and ^**^*p*<0.01 vs. WT 30°C.


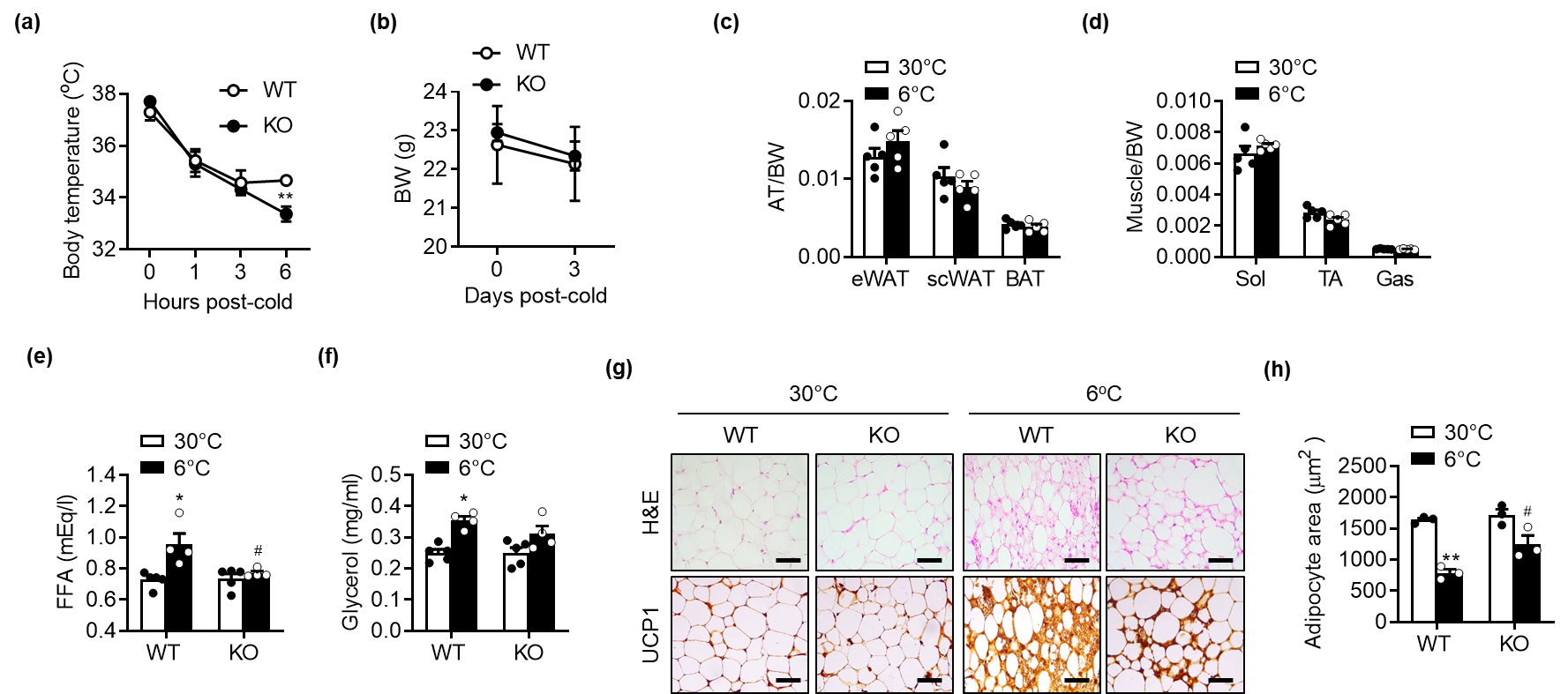


**Figure S4. Impairment of adaptive thermogenesis and subcutaneous fat beiging in female mS6KO mice after cold exposure.** Eight-week-old female mS6KO mice and their wild-type (WT) littermates were housed at 30°C for 1 day and then exposed to cold (6°C). (a) Rectal temperature of mice after cold exposure (n=5). (b) Body weights of mice housed at 6°C for three days (n=5). (c, d) Weights of adipose tissue (AT), soleus (Sol), tibialis anterior (TA), gastrocnemius (Gas) muscles as percentages of body weight (BW) after 3 days of cold exposure (n=5). (e, f) Serum levels of free fatty acid (FFA) and glycerol were measured (n=4-5). (g) scWAT was H&E stained or immunostained with an antibody against UCP1. Bars=25 μm. (h) Cell size analysis of adipocytes from scWAT was carried out on two sections per mouse.

**
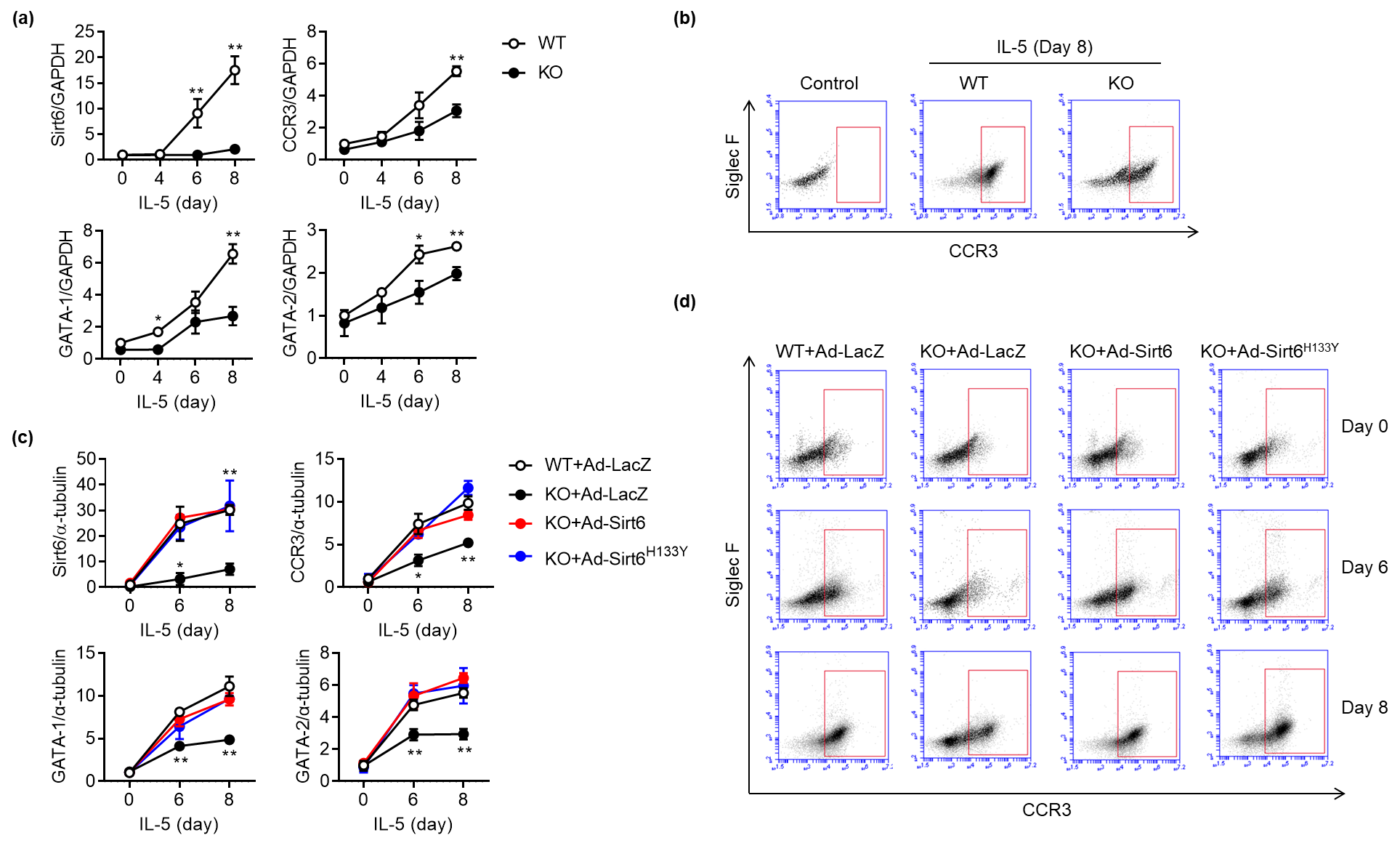
 Figure S5. Quantification in ex vivo eosinophil cultures and representative flow cytometry plots.** All experimental procedures were as shown in Figure 5. (a, b) BMCs from WT or mS6KO mice following treatment with IL-5 were prepared and analyzed for specific genes by western blotting (quantification results are shown) or by flow cytometry. Representative flow cytometry plots showing the gating strategy for identifying eosinophils. (c, d) BMCs from WT or mS6KO mice were transduced with Ad-LacZ, Ad-Sirt6, or Ad-mSirt6 and treated with 10 ng/ml IL-5 for 8 days. Quantification results of western blotting and representative flow cytometry plots are shown.


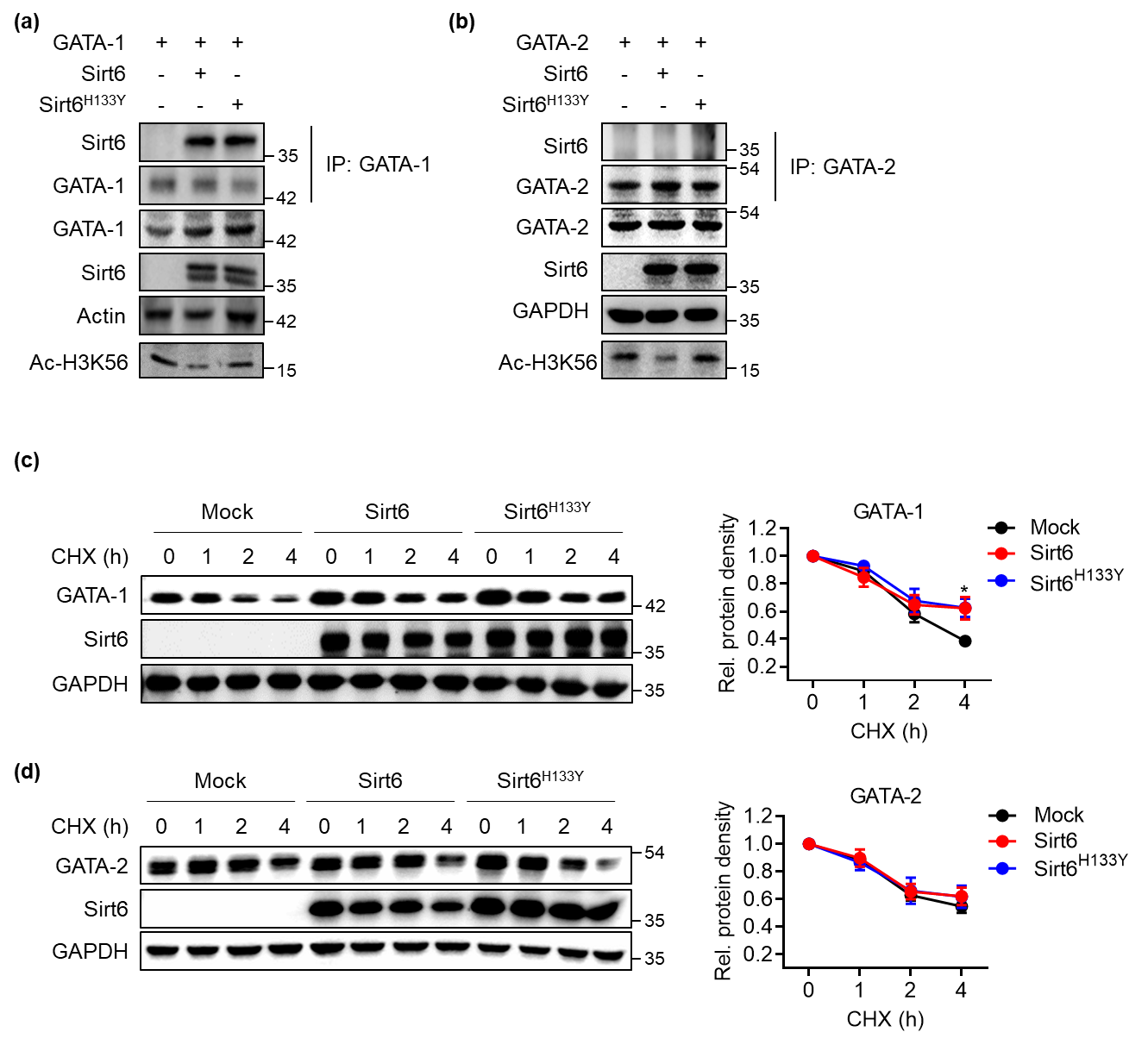


**Figure S6. GATA-1 protein stabilization by Sirt6.** (a, b)HEK293T cells were transfected with plasmid encoding either GATA-1 or GATA-2 with Sirt6 and deacetylase mutated Sirt6 (mSirt6) as indicated. Anti-GATA-1 and anti-GATA-2 immunoprecipitates were immunoblotted with anti-Sirt6 antibodies. (c, d) HEK293T cells transfected with mock or plasmid encoding Sirt6 or mSirt6 in combination with GATA-1 or GATA-2 were treated with cycloheximide (CHX, 20 μg/ml) for indicated time periods, and relative protein levels of GATA-1 or GATA-2 were compared (n=4). Values are the mean ± SEM. ^*^*p*<0.05 vs. mock.

**
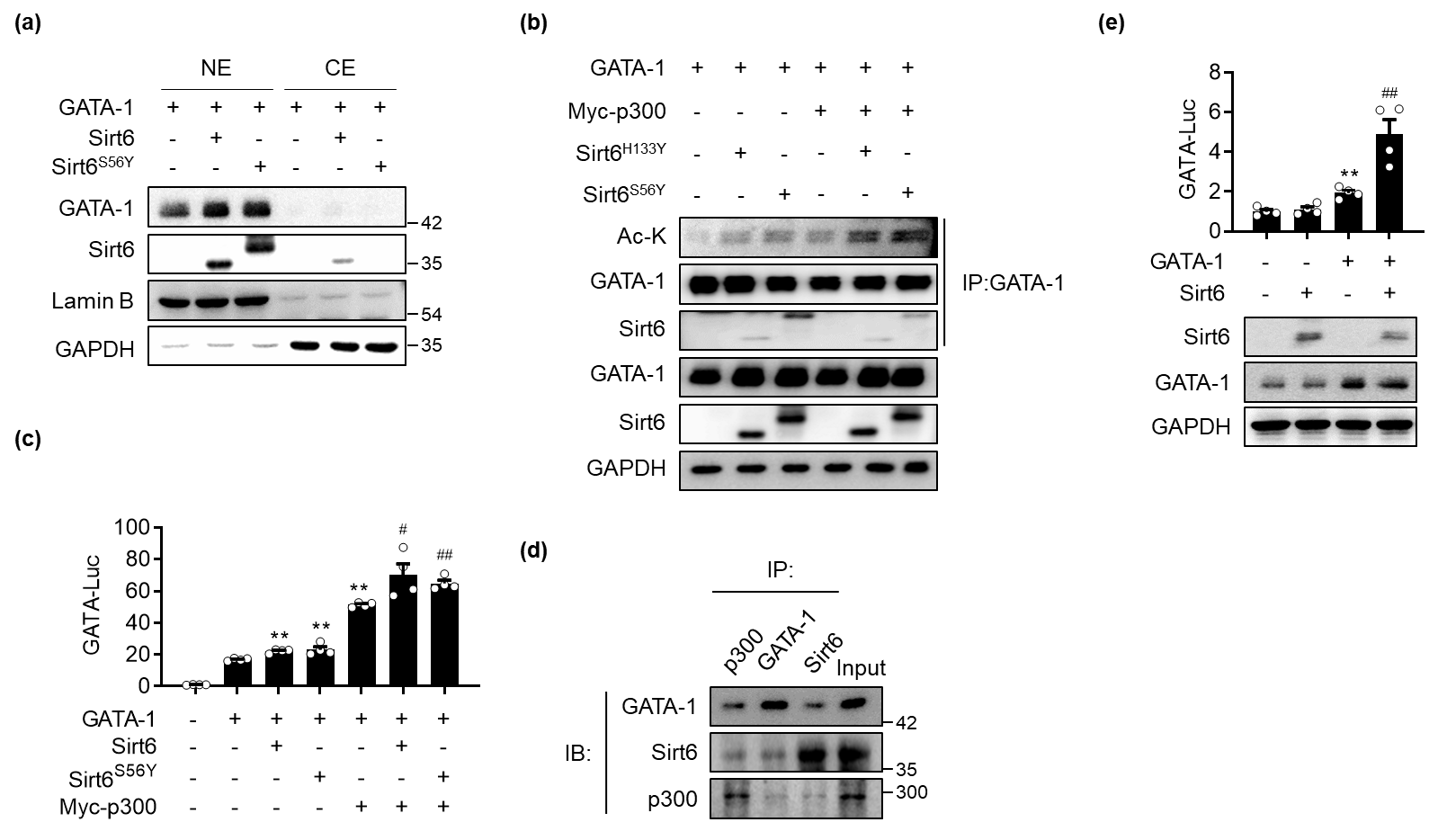
**

**Figure S7. Enhancement of GATA-1 transcriptional activity by Sirt6^S56Y^, and Sirt6 regulation of GATA-1 in AML14.3D10 cell lines.** (a) HEK293T cells were transfected with plasmid encoding GATA-1, Sirt6, and deacetylase and ADP-ribosyltransferase mutated Sirt6 (Sirt6^S56Y^) as indicated. GATA-1 protein levels in the nuclear extract (NE) and cytoplasmic extract (CE) were measured. (b) HEK293T cells were transfected with indicated plasmids and total cell lysates were used for co-IP experiments. (c) After transfection in HEK293T cells as indicated, the relative GATA-1 luciferase activity in the cell lysates was measured (n=4). (d, e) Physical interaction among GATA-1, Sirt6, and p300 and the enhancement of GATA-1 transcriptional activity by Sirt6 were confirmed in a human eosinophil cell line AML14.3D10. Values are the mean±SEM. ^**^*p*<0.01 vs GATA-1; ^#^*p*<0.05 and ^##^*p*<0.01 vs. GATA-1+p300.
